# Supplementary material for: Alcohol Use and the Risk of Colorectal Liver Metastasis: A Systematic Mapping Review
Source: Biology (Basel). 2023 Feb 6;12(2):257. doi: 10.3390/biology12020257 (PMC9953220; doi:10.3390/biology12020257)
Supplement: Supplementary file 1 [file biology-12-00257-s001.zip › biology-2176418-supplementary.pdf]

File S1: Supplementary Material (Methods: Mapping Review Search Strategy)

The following strategy was applied for PubMed: ((Alcohol*[tiab] OR binge[tiab] OR binging[tiab] OR "Alcohol Drinking"[Mesh] OR "Alcoholism"[Mesh] OR "Binge Drinking"[Mesh]) OR (Alcohol*[tiab] AND (hepatic[tiab] OR hepatob*[tiab] OR hepatoc*[tiab] OR hepatitis[tiab] OR "Hepatitis"[Mesh] OR fatty OR hepatit* OR liver[tiab] OR "Liver"[Mesh])) OR "Liver Diseases, Alcoholic"[Mesh] OR "Fatty Liver, Alcoholic"[Mesh]) AND (((hepatic[tiab] OR hepatob*[tiab] OR hepatoc*[tiab] OR liver[tiab] OR "Liver"[Mesh]) AND (CRLM OR "secondary"[Subheading] OR secondary[tiab] OR secondarily[tiab] OR reoccurrence* OR recurrence* OR metasta* OR "Neoplasm Metastasis"[Mesh])) OR "Liver Neoplasms/secondary"[Mesh]) AND (((colorectal[tiab] OR colon [tiab] OR rectum [tiab] OR intestin* [tiab] OR anal [tiab] OR "Intestine, Large"[Mesh] AND (malignan*[tiab] OR carcinoma*[tiab] OR "Carcinoma"[Mesh] OR cancer[tiab] OR cancers[tiab] OR neoplas*[tiab])) OR "Colorectal Neoplasms"[Mesh] OR "Intestinal Neoplasms"[Mesh])) AND ("Case-Control Studies"[Mesh] OR “case control” OR “case controlled” OR risk[tiab] OR "Risk"[Mesh] OR “risk”[tiab] OR risks[tiab] OR incidence[tiab] OR "Incidence"[Mesh] OR "Epidemiology"[Mesh] OR epidemiolog*[tiab] OR "epidemiology" [Subheading] OR "Longitudinal Studies"[Mesh] OR longitudinal*[tiab] OR "Follow-Up Studies"[Mesh] OR “follow-up”[tiab] OR "Prospective Studies"[Mesh] OR prospectiv*[tiab] OR retrospective*[tiab]) NOT (non-alcoholic NOT alcoholic).

For Embase, the following algorithm was used: ('drinking behavior'/exp OR 'binge drinking'/exp OR (alcohol* OR binge OR binging):ti,ab,kw OR (Alcohol*:ti,ab,kw AND (hepatic OR hepatob* OR hepatoc* OR hepatitis:ti,ab,kw OR ‘hepatitis’/exp OR fatty OR hepatit* OR liver)) OR 'alcoholic fatty liver'/exp) AND (('liver cancer'/exp OR 'liver'/exp OR liver:ti,ab,kw) AND (CRLM OR 'metastasis'/exp OR 'cancer recurrence'/exp OR reoccurrence* OR recurrence* OR secondary OR secondarily)) AND ('colorectal cancer'/exp OR 'rectum cancer'/exp OR 'intestine cancer'/exp OR 'colon cancer'/exp OR 'anus cancer'/exp OR ((colon:ti,ab,kw OR colorectal:ti,ab,kw) AND democarcinoma:ti,ab,kw)) AND (risk:ti,ab,kw OR risks:ti,ab,kw OR 'case control study'/exp OR ‘case?control*’ OR incidence:ti,ab,kw OR 'cancer incidence'/exp OR 'epidemiology'/exp OR epidemiology:ti,ab,kw OR 'longitudinal study'/exp OR longitudial*:ti,ab,kw OR 'follow up'/exp OR follow?up:ti,ab,kw OR 'prospective study'/exp OR prospectiv*:ti,ab,kw) NOT (‘non-alcoholic’ OR 'nonalcoholic') NOT 'alcoholic').
